# New Insights into Diagnosis and Treatment of Renal Cell Carcinoma, Bladder Cancer, and Prostate Cancer

**DOI:** 10.1155/2017/6467072

**Published:** 2017-03-13

**Authors:** Piotr L. Chlosta, Tomasz Golabek, Péter Nyirády

**Affiliations:** ^1^Department of Urology, Jagiellonian University in Krakow, ul. Grzegorzecka 18, 31-531 Krakow, Poland; ^2^Department of Urology and Centre for Urooncology, Semmelweis University, Üllői út 78/b, Budapest 1082, Hungary

In recent years, substantial changes in urological cancer-related mortality have occurred. These have resulted from therapeutic improvements of prostatic cancer, decreased exposure to tobacco smoking, and occupational carcinogens of bladder and possibly kidney cancers. Despite improved primary prevention, detection, and treatment, the incidence of age-related cancers of the urinary tract is likely to rise as a result of global population ageing. Therefore, it is vital to identify and address the most relevant targets for further early detection, investigation, and therapy of urological malignancies.

In keeping with this spirit, this special issue brings articles that investigated clinical and prognostic significance of several factors in the three most common urological cancers: renal cell carcinoma, prostate cancer, and bladder cancer.

G. Ji et al. in their report analysed pathological features of 2,929 men diagnosed with prostate cancer within different age groups including patients older than 75 years of age. They found that both patients aged ≤55 years and >75 years are more likely to be diagnosed with more aggressive disease. These findings have certain consequences including more aggressive treatment of the disease also in elderly healthy men and bring us into opposition with supporters of nonradical management of prostate cancer in older men.

Two research articles are dedicated to the prognostic role of blood-derived factors in patients with renal cell carcinoma. Y. Tian et al. in their systematic review and meta-analysis provide an evidence for elevated plasma fibrinogen to be adversely associated with overall, cancer-specific, and disease-free survival. S.-S. Byun et al. assessed the prognostic significance of preoperative neutrophil-to-lymphocyte ratio in nonmetastatic renal cell carcinoma. Their findings showed that the investigated parameter was associated with worse clinical tumour behavior, and it was a significant prognostic factor for both recurrence-free and cancer-specific survival in that group of patients.

Predictors of short- and long-term deterioration in renal function after partial nephrectomy in patients with renal cell carcinoma or benign tumour with or without preoperative predisposition to chronic kidney disease were studied by S. H. Kim et al. Their findings confirmed our understanding that abnormal preoperative renal function is associated with long-term deterioration of renal function and also indicated the baseline state of the renal function as the predominant factor affecting the postoperative functional outcome more than other determinants including partial nephrectomy procedure or renal cell carcinoma itself.

Urothelial bladder cancer remains a lethal malignancy in a significant proportion of advanced cases; thus more useful and reliable biomarkers that provide additional prognostic information are needed. In the quest for the better prognosticator in that group of patients, for the first time S. Ohtake et al. evaluated an impact of neutrophil-to-lymphocyte ratio in patients with advanced bladder cancer who received gemcitabine and nedaplatin therapy. Their findings suggest that this simple biomarker may serve as a new biomarker to predict responses to chemotherapy in advanced bladder cancer patients.

*Piotr L. Chlosta* *Tomasz Golabek* *Péter Nyirády*

